# Sarcopenia as a Predictor of Mortality Among Cirrhotic Patients Awaiting Liver Transplantation

**DOI:** 10.1155/ijh/3205201

**Published:** 2026-07-29

**Authors:** Sorachat Niltwat, Pornpim Korpraphong, Khemajira Karaketklang, Phunchai Charatcharoenwitthaya

**Affiliations:** ^1^ Division of Gastroenterology, Department of Medicine, Faculty of Medicine Siriraj Hospital, Mahidol University, Bangkok, Thailand, mahidol.ac.th; ^2^ Department of Radiology, Faculty of Medicine Siriraj Hospital, Mahidol University, Bangkok, Thailand, mahidol.ac.th

**Keywords:** Child–Turcotte–Pugh, liver transplantation, MELD-Na, mortality, sarcopenia

## Abstract

**Background and Aims:**

Sarcopenia is common among cirrhotic patients awaiting liver transplantation, but its prognostic significance in the Asian population remains unclear. This retrospective study aimed to evaluate the association between sarcopenia and mortality risk in Thai cirrhotic patients awaiting transplantation.

**Patients and Methods:**

We analyzed data from 162 cirrhotic patients (112 men; mean age 55.0 ± 8.2 years) listed for liver transplantation with available computed tomography for skeletal muscle index (SMI) measurement at the third lumbar vertebra. Sarcopenia was defined using sex‐specific thresholds by the Japan Society of Hepatology. Waitlist mortality was analyzed using the Fine–Gray competing risk model, with liver transplantation treated as a competing event.

**Results:**

The mean SMI was 45.6 ± 9.1 cm^2^/m^2^, with 27.8% of patients having sarcopenia. Cirrhosis etiology was not associated with sarcopenia. During a median waitlist time of 13.5 months, 87 patients (53.7%) died while waiting, and 45 (27.8%) were transplanted. Sarcopenic patients had a shorter median transplant‐free survival (8.4 vs. 16.7 months, *p* = 0.029) and were more prone to bacterial infections (51.1% vs. 34.2%, *p* = 0.049) and sepsis‐related death (33.3% vs. 17.9%, *p* = 0.036). Sarcopenia significantly increased the risk of death on the waiting list (subdistribution hazard ratio [sHR] 1.72; 95% CI 1.12‐2.66), particularly in patients with Child–Turcotte–Pugh class A/B cirrhosis (sHR 2.38; 95% CI 1.38–4.11). In multivariate analysis, sarcopenia remained an independent predictor of mortality (adjusted sHR 1.69; 95% CI 1.04–2.76) after adjusting for sex and Model for End‐Stage Liver Disease–sodium score.

**Conclusion:**

Sarcopenia is a significant predictor of mortality in cirrhotic patients awaiting liver transplantation.

## 1. Introduction

Cirrhosis represents the end stage of chronic liver diseases and carries a 5‐year mortality rate ranging from 2% to 88%, depending upon the severity of liver dysfunction [1]. Prognostic assessment in cirrhotic patients is consequently an essential part of care provision with the intention of identifying and adjudicating timely intervention to those who are most at risk of decompensating events and mortality. The Child–Turcotte–Pugh (CTP) score and Model for End‐Stage Liver Disease (MELD) score have been extensively utilized in routine practice since their inception to determine prognosis in cirrhotic patients due to their simplicity based on hepatic dysfunction parameters [2–5]. However, they are not without inherent limitations. The CTP score includes subjective components such as degrees of ascites accumulation and hepatic encephalopathy, which are prone to interobserver variability and may be affected by treatment. In contrast, the MELD score has been reported to misclassify prognosis in 15%–20% of cirrhotic patients [6].

Nutritional status emerges as a critical yet often overlooked prognostic factor for adverse clinical outcomes in cirrhotic patients. Individuals with cirrhosis are predisposed to malnutrition due to various factors such as reduced oral intake stemming from dysgeusia, early satiety from ascites, fasting for procedures, or decompensating events (e.g., variceal bleeding, hepatic encephalopathy), alongside maldigestion, malabsorption, and high energy expenditure resulting from chronic inflammation, hypercatabolic state, and accelerated starvation [7]. Several studies have revealed that sarcopenia, defined as a state of reduced skeletal muscle mass associated with malnutrition, prevails in 40%–70% of cirrhotic patients [8, 9]. The presence of sarcopenia in cirrhosis has been associated with reduced quality of life [10], prolonged hospital stay [11], and increased risks of infection [12], hepatic decompensation [13, 14], and mortality [15, 16]. However, assessing malnutrition, particularly sarcopenia, in cirrhotic patients is challenging due to the limited utility of conventional tools such as subjective global assessment, body mass index (BMI), anthropometry, mid‐upper arm circumference, bioelectrical impedance analysis, and dual‐energy X‐ray absorptiometry, owing to their subjective nature, interobserver variability, and interference by fluid retention [9, 17, 18].

Cross‐sectional imaging with computed tomography (CT) has emerged as an objective and well‐validated method for quantifying skeletal muscle index (SMI) in cirrhotic patients [7, 16]. This technique minimizes the influence of fluid retention and reduced physical activity [19], and its frequent use for clinical assessment, especially during transplant evaluation for hepatocellular carcinoma (HCC), ensures accessibility, reproducibility, and practical relevance in routine care. The presence of sarcopenia, initially defined by SMI at the third lumbar vertebra (L3) [20], has been shown to be a robust predictor of overall mortality among patients on transplant waitlists [21]. However, its prognostic value may potentially be influenced by regional practices as well as transplant rates [22]. Concerns also exist regarding the application and validity of cutoff optimums across different races and ethnicities, particularly among Asians, where skeletal muscle mass is estimated to be 15%–17% lower than that of Caucasians [23–27]. Therefore, this study aimed to determine the prevalence of sarcopenia and its prognostic value in predicting mortality among Thai cirrhotic patients awaiting liver transplantation.

## 2. Patients and Methods

### 2.1. Study Population

In this retrospective study, we analyzed data from patients registered on the liver transplant waiting list at the Faculty of Medicine Siriraj Hospital, Bangkok, Thailand, between 2005 and 2013. Eligible patients were aged 18 years or older and had a diagnosis of cirrhosis confirmed through clinical, biochemical, and imaging assessments; had undergone a liver transplantation evaluation; and were subsequently listed for transplantation. These patients had CT images and laboratory results available from the time of evaluation. The abdominal CT scans were performed for assessing biliary and vascular anatomy. We excluded patients with active malignancies other than HCC, acute liver failure, acute‐on‐chronic liver failure, or significant comorbidities that could contribute to sarcopenia or increase mortality risk, including end‐stage renal disease requiring dialysis, chronic obstructive pulmonary disease requiring oxygen supplementation, and congestive heart failure.

### 2.2. Clinical and Laboratory Assessment

Clinical data extracted from medical records included age, sex, weight, height, BMI, comorbidities, etiology of cirrhosis, and history of cirrhotic complications such as ascites, spontaneous bacterial peritonitis, refractory ascites, hepatic encephalopathy, and hepatorenal syndrome, as well as episodes of infection and antimicrobial treatment. Blood chemistries, prothrombin time, international normalized ratio (INR), CTP score, original MELD score, and MELD‐sodium (MELD‐Na) score were obtained from the data collected at the time of the index CT scan, conducted for liver transplant evaluation. The diagnosis of HCC was made according to the international HCC guidelines, which involve identifying typical lesions on dynamic imaging studies or obtaining histologic confirmation [28].

As part of our practice, cirrhotic patients awaiting liver transplantation underwent HCC surveillance and received specific therapy for underlying liver cirrhosis (such as oral antiviral agents for hepatitis B virus [HBV] infection, immunosuppressive drugs for autoimmune liver disease), non‐selective beta blockers for primary or secondary prophylaxis of variceal bleeding, and diuretics for managing ascites.

### 2.3. Definition of Sarcopenia

Skeletal muscle mass was assessed by measuring the cross‐sectional areas of skeletal muscles (cm^2^) at the L3. This measurement was performed using a specialized workstation designed for tissue quantification (GE Healthcare, UK). The Hounsfield unit (HU) range for identifying skeletal muscle was set between −30 and 150 HU. The muscles evaluated included the psoas, paraspinal, transversus abdominis, rectus abdominis, quadratus lumborum, and internal and external obliques in the specified axial images. The measured skeletal muscle tissue area was then adjusted for height to normalize for body habitus (cm^2^/m^2^), resulting in the SMI [21, 29–31]. Sarcopenia was defined using cutoff values of 42 cm^2^/m^2^ for men and 38 cm^2^/m^2^ for women according to the Japan Society of Hepatology guidelines for sarcopenia in liver disease [24].

### 2.4. Ethics Statement

This study was approved by the Institutional Review Board of the Faculty of Medicine Siriraj Hospital (COA No. Si547/2013) and was performed according to the Declaration of Helsinki. Informed consent was waived by the Ethics Committee due to the retrospective design of the study.

### 2.5. Statistical Analysis

Continuous variables were expressed as mean ± standard deviation or median (range), as appropriate. Comparisons between groups were performed using the *t*‐test or Mann–Whitney *U* test for continuous variables and the Chi‐square test or Fisher′s exact test for categorical variables. Correlations between SMI and liver disease severity scoring systems were evaluated using the Spearman correlation coefficient.

Patient survival was evaluated from the date of the index CT (time zero), performed as part of the liver transplant assessment, until death, liver transplantation, or the last follow‐up, whichever came first. Overall transplant‐free survival was estimated using the Kaplan–Meier method and compared using the log‐rank test.

Because liver transplantation could preclude the occurrence of waiting list mortality, transplantation was treated as a competing event in competing risk analyses. The association between sarcopenia and waitlist mortality was therefore assessed using the Fine–Gray subdistribution hazards model, and results were reported as subdistribution hazard ratios (sHRs) with corresponding 95% confidence intervals (CIs). Variables considered clinically relevant or significant in univariate analysis without evidence of collinearity were entered into multivariable competing risk regression models. Candidate variables included age, sex, BMI, cirrhosis etiology, history of cirrhotic complications, liver biochemistries, INR, creatinine, serum sodium, CTP score, original MELD score, MELD‐Na score, and sarcopenia. A forward stepwise likelihood ratio approach was used to reduce overfitting and retain variables with independent prognostic relevance. Interaction analyses were additionally performed within the Fine–Gray competing risk framework to evaluate potential effect modification according to cirrhosis etiology and coexisting alcohol‐related or metabolic features. All statistical tests were two‐tailed, with a significance level of *α* = 0.05. Statistical analyses were performed using STATA version 14.0 (StataCorp LP, College Station, Texas, USA).

## 3. Results

### 3.1. Characteristics of the Study Population

The characteristics of the study cohort are outlined in Table 1. The cohort comprised 162 patients with cirrhosis awaiting liver transplantation, of whom 112 (69.1%) were male. The mean age of the patients was 55.0 ± 8.2 years, with a mean BMI of 24.9 ± 4.1 kg/m^2^. The etiology of cirrhosis was distributed as follows: HBV (35.2%), hepatitis C virus (HCV) (25.9%), alcohol‐associated liver disease (10.5%), autoimmune liver disease (6.8%), alcohol in combination with HBV or HCV (9.3%), metabolic dysfunction–associated steatotic liver disease (3.1%), HBV and HCV coinfection (2.5%), and others (6.8%).

**Table 1:** Baseline characteristics of the study population.

Characteristics	Total cohort (*n* = 162)	Sarcopenia (*n* = 45)	No sarcopenia (*n* = 117)	*p* value
Age, years	55.0 ± 8.2	56.4 ± 9.8	54.5 ± 7.4	0.245
Male gender, *n* (%)	112 (69.1)	30 (66.7)	82 (70.1)	0.674
BMI, kg/m^2^	24.9 ± 4.1	21.9 ± 3.2	26.1 ± 3.8	< 0.001
Comorbidities, *n* (%)				
Cardiovascular disease	2 (1.2)	1 (2.2)	1 (0.9)	0.480
Diabetes mellitus	60 (37.0)	13 (28.9)	47 (40.2)	0.184
Hypertension	48 (29.6)	9 (20.0)	39 (33.3)	0.097
Dyslipidemia	20 (12.3)	4 (8.9)	16 (13.7)	0.408
Chronic kidney disease	4 (2.5)	2 (4.4)	2 (1.7)	0.317
Etiology of cirrhosis, *n* (%)				0.343
Hepatitis B virus	57 (35.2)	16 (35.6)	41 (35.0)	
Hepatitis C virus	42 (25.9)	7 (15.6)	35 (29.9)	
Alcohol	17 (10.5)	7 (15.6)	10 (8.5)	
Alcohol and hepatic B/C virus	15 (9.3)	3 (6.7)	12 (10.2)	
Autoimmune liver disease	11 (6.8)	5 (11.1)	6 (5.1)	
MASLD	5 (3.1)	2 (4.4)	3 (2.6)	
Hepatitis B and C virus coinfection	4 (2.5)	1 (2.2)	3 (2.6)	
Others	11 (6.8)	4 (8.8)	7 (6.0)	
History of cirrhotic complications, *n* (%)				
Hepatic encephalopathy	38 (23.5)	15 (33.3)	23 (19.7)	0.067
Variceal bleeding	39 (24.1)	14 (31.1)	25 (21.4)	0.195
Ascites	72 (44.4)	28 (62.2)	44 (37.6)	0.005
Spontaneous bacterial peritonitis	24 (14.8)	10 (22.2)	14 (12.0)	0.101
Refractory ascites	10 (6.2)	5 (11.1)	5 (4.3)	0.106
Hepatorenal syndrome	5 (3.1)	1 (2.2)	4 (3.4)	0.694
HCC at the time of waiting list	64 (39.5)	20 (44.4)	44 (37.6)	0.427
Laboratory				
AST, U/L	64 (44–190)	62 (42–138)	65 (44–110)	0.619
ALT, U/L	39 (28–120)	45 (25–67)	39 (29–72)	0.486
Total bilirubin, mg/dL	2.2 (1.2–25.5)	2.6 (1.6–5.6)	1.8 (1.0–3.2)	0.004
Albumin, g/dL	3.1 ± 0.6	3.0 ± 0.6	3.2 ± 0.6	0.038
Globulin, g/dL	4.4 ± 1.0	4.2 ± 0.9	4.4 ± 1.1	0.359
INR	1.5 ± 0.5	1.5 ± 0.4	1.5 ± 0.5	0.518
Creatinine, mg/dL	0.9 (0.7–2.0)	0.8 (0.7–1.0)	0.9 (0.7–1.1)	0.050
Sodium, mmol/L	135.7 ± 4.4	134.9 ± 4.7	136.1 ± 4.3	0.129
Child–Turcotte–Pugh score	8.3 ± 2.4	9.2 ± 2.5	8.0 ± 2.3	0.002
Child–Turcotte–Pugh class, *n* (%)				0.007
A	47 (29.0%)	6 (13.3)	41 (35.0)	
B	61 (37.7%)	17 (37.8)	44 (37.6)	
C	54 (33.3%)	22 (48.9)	32 (27.4)	
MELD score	14.9 ± 6.6	15.8 ± 5.8	14.6 ± 6.9	0.271
MELD‐Na score	15.9 ± 7.9	17.4 ± 7.0	15.2 ± 8.3	0.138

*Note:* Results presented as mean ± standard deviation, median (interquartile range), or number (percentage).

Abbreviations: ALT, alanine aminotransferase; AST, aspartate aminotransferase; BMI, body mass index; INR, international normalized ratio; MASLD, metabolic dysfunction–associated steatotic liver disease; MELD, Model for End‐stage Liver Disease; MELD‐Na, Model for End‐stage Liver Disease–sodium.

### 3.2. Prevalence and Features Associated With Sarcopenia

The mean SMI was 45.6 ± 9.1 cm^2^/m^2^ (range, 25.1–71.3), and sarcopenia was present in 45 patients (27.8%). The prevalence of sarcopenia did not differ significantly between patients with and without HCC at the time of listing in the overall cohort (44.4% vs. 37.6%, *p* = 0.427) or within the HBV/HCV‐related cirrhosis subgroup (29.5% vs. 18.6%, *p* = 0.198).

Patients with sarcopenia had significantly lower BMI (21.9 ± 3.2 vs. 26.1 ± 3.8 kg/m^2^, *p* < 0.001) and SMI (35.3 ± 4.5 vs. 49.6 ± 7.1 cm^2^/m^2^, *p* < 0.001) compared with those without sarcopenia. The prevalence of comorbidities, including atherosclerotic cardiovascular disease, diabetes mellitus, hypertension, dyslipidemia, and chronic kidney disease, was similar between groups. Patients with sarcopenia tended to have a history of hepatic encephalopathy (33.3% vs. 19.7%, *p* = 0.067). They also more frequently had a history of ascites and exhibited higher serum total bilirubin levels, along with lower serum albumin and creatinine levels. No differences were observed in demographics or the etiology of underlying liver disease between cirrhotic patients with and without sarcopenia, as shown in Table 1.

Patients with sarcopenia demonstrated more advanced liver disease, as evidenced by higher CTP scores and classifications. An inverse correlation was observed between SMI and the CTP score (*r* = −0.165, *p* = 0.036). However, there were no differences in MELD and MELD‐Na scores between cirrhotic patients with and without sarcopenia. In addition, no significant correlations were identified between SMI and the MELD score (*r* = 0.014, *p* = 0.860) or the MELD‐Na score (*r* = −0.018, *p* = 0.828).

### 3.3. Mortality of Patients With and Without Sarcopenia

**Figure 1:**
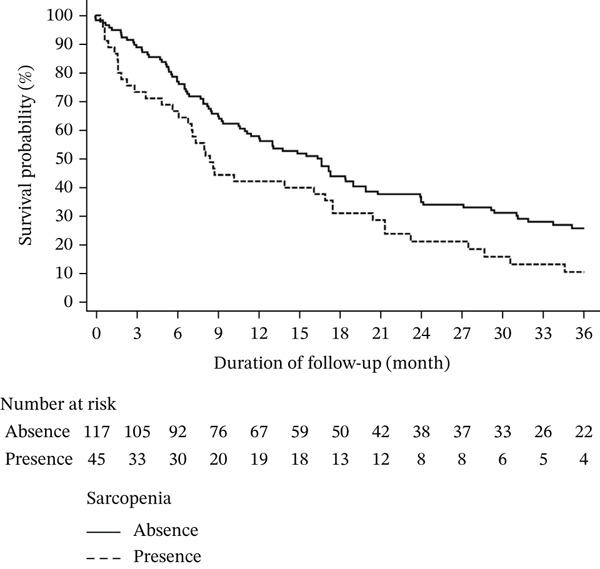
Kaplan–Meier curves of transplant‐free survival among cirrhotic patients awaiting liver transplantation according to sarcopenia status.

During a median waitlist time of 13.5 months (interquartile range, 6.0–29.1 months), 87 (53.7%) patients died without liver transplantation, whereas 45 (27.8%) patients were transplanted. Among the deceased patients, 36 (40.4%) died from sepsis, 23 (25.8%) with HCC died from hepatic decompensation, 16 (18.0%) died from progressive liver failure with multiorgan failure, six (6.7%) from variceal bleeding, three (3.4%) from intracerebral hemorrhage, two (2.2%) from acute renal failure, and one (1.1%) from a cardiovascular event. Of these, 18 deaths occurred within 3 months after being placed on the waiting list. Median transplant‐free survival was 8.4 months (95% CI, 6.1–16.9) in patients with sarcopenia and 16.7 months (95% CI, 11.2–19.0) in those without sarcopenia (*p* = 0.029), as illustrated in Figure 1. The overall probability of transplant‐free survival at 3 months was 73.3% and 90.5% in cirrhotic patients with and without sarcopenia, respectively.

**Figure 2:**
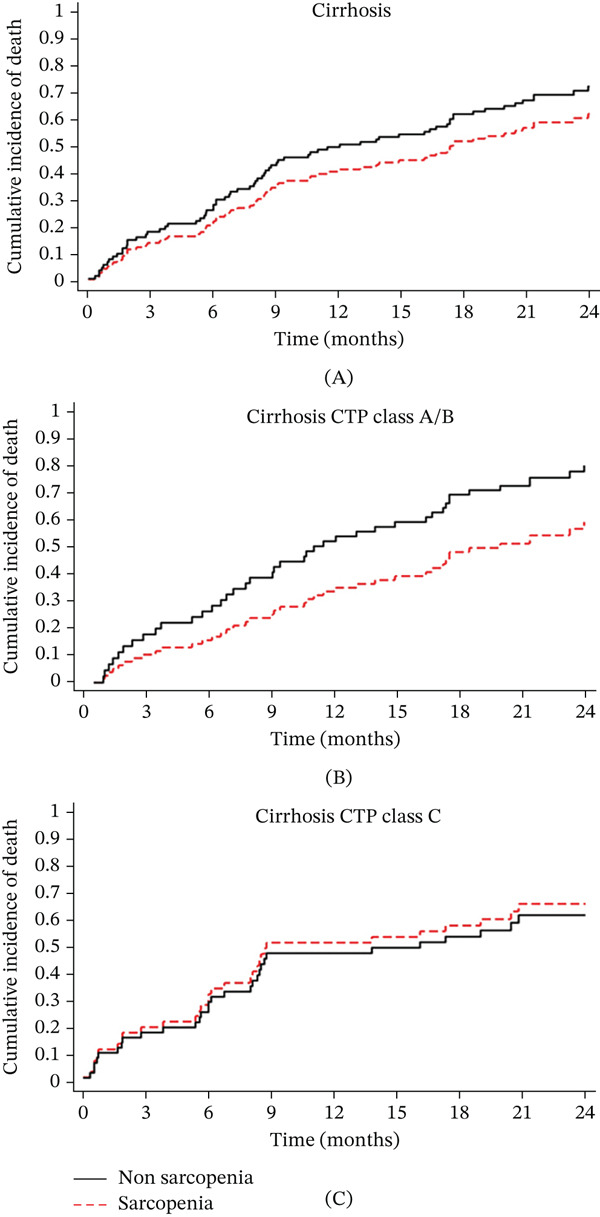
Cumulative incidence of waitlist mortality according to sarcopenia status. The curves illustrate the cumulative incidence of death among (A) the total cohort of cirrhotic patients, (B) patients with Child–Turcotte–Pugh (CTP) class A/B, and (C) patients with CTP class C cirrhosis. These estimates were calculated using the Fine–Gray model, treating liver transplantation as a competing event. The solid lines represent patients with sarcopenia, and the dashed lines represent patients without sarcopenia.

Figure 2A–C shows cumulative incidence curves comparing waitlist mortality between cirrhotic patients with and without sarcopenia. Overall, sarcopenia was associated with a significantly higher risk of waitlist mortality (sHR, 1.72; 95% CI, 1.12–2.66; *p* = 0.014) (Figure 2A). Among patients with CTP class A/B cirrhosis, sarcopenia was associated with a markedly increased risk of death (sHR, 2.38; 95% CI, 1.38–4.11; *p* = 0.002) (Figure 2B), whereas no significant difference was observed in those with CTP class C cirrhosis (sHR, 0.89; 95% CI, 0.45–1.77; *p* = 0.742) (Figure 2C).

In exploratory subgroup analyses, sarcopenia was associated with increased waitlist mortality in 118 patients with HBV/HCV‐related cirrhosis (65 deaths; 31 transplantations; sHR, 2.30; 95% CI, 1.39–3.79; *p* = 0.001), whereas no significant association was observed in 44 patients with nonviral‐related cirrhosis (22 deaths; 14 transplantations; sHR, 0.97; 95% CI, 0.42–2.21; *p* = 0.938). The interaction between sarcopenia and cirrhosis etiology was not statistically significant (*p* for interaction = 0.647). Additional exploratory analyses were performed within the HBV/HCV subgroup according to the presence of coexisting alcohol‐related or metabolic features. Among 54 patients without alcohol use or metabolic features (32 deaths; 13 transplantations), sarcopenia was associated with increased waitlist mortality (sHR, 2.51; 95% CI, 1.28–4.90; *p* = 0.007). Among 64 patients with coexisting alcohol‐related or metabolic features (33 deaths; 18 transplantations), the association did not reach statistical significance (sHR, 2.03; 95% CI, 0.97–4.28; *p* = 0.062). The interaction between sarcopenia and the presence of alcohol‐related or metabolic features was not statistically significant (*p* for interaction = 0.738).

During the wait for liver transplantation, bacterial infection developed more frequently in cirrhotic patients with sarcopenia than those without sarcopenia (51.1% vs. 34.2%, *p* = 0.049). Consequently, patients with sarcopenia were more likely to die from sepsis than those without sarcopenia (33.3% vs. 17.9%, *p* = 0.036). No significant differences were found in other causes of death between cirrhotic patients with and without sarcopenia. In subgroup analyses, bacterial infections were more frequent in sarcopenic patients with HBV/HCV‐related cirrhosis compared with those without sarcopenia (62.5% vs. 39.2%, *p* = 0.046), whereas no significant difference was observed in nonviral‐related cirrhosis (38.1% vs. 23.7%, *p* = 0.246).

### 3.4. Predictors of Mortality in Cirrhotic Patients Waiting for Liver Transplantation

Univariate Fine–Gray competing risk analysis revealed that male gender (*p* = 0.049), a history of hepatic encephalopathy (*p* < 0.001), as well as biochemistries including the AST/ALT ratio (*p* = 0.007), INR (*p* < 0.001), creatinine (*p* = 0.011), and sodium (*p* = 0.050), and the severity of liver disease as indicated by the CTP score (*p* = 0.017), MELD score (*p* = 0.025), MELD‐Na score (*p* = 0.016), and sarcopenia (*p* = 0.014), were significantly associated with the risk of death among patients awaiting liver transplantation (Table 2). In the multivariate competing risk analysis, sarcopenia remained significantly associated with an increased mortality risk (adjusted sHR, 1.69; 95% CI, 1.04–2.76, *p* = 0.033) after adjusting for sex and the MELD‐Na score.

**Table 2:** Factors associated with waitlist mortality among cirrhotic patients awaiting liver transplantation.

Variable	Univariate analysis		Multivariate analysis	
	SHR (95% CI)	*p* value	SHR (95% CI)	*p* value
Age, years	1.00 (0.97–1.03)	0.812		
Male gender	1.63 (1.03–2.66)	0.049	1.63 (0.95–2.79)	0.073
BMI, kg/m^2^	0.97 (0.92–1.02)	0.217		
Alcohol‐related cirrhosis	1.11 (0.68–1.80)	0.671		
HBV‐infected cirrhosis	0.93 (0.60–1.43)	0.728		
HCV‐infected cirrhosis	1.13 (0.75–1.71)	0.571		
HBV/HCV‐related cirrhosis	0.98 (0.63–1.51)	0.916		
History of cirrhotic complications				
Hepatic encephalopathy	2.88 (1.75–4.71)	< 0.001		
Variceal bleeding	1.17 (0.71–1.92)	0.538		
Ascites	1.34 (0.88–2.05)	0.180		
Spontaneous bacterial peritonitis	1.33 (0.69–2.56)	0.402		
Refractory ascites	1.62 (0.76–3.49)	0.215		
Hepatorenal syndrome	2.99 (0.81–11.03)	0.099		
HCC at the time of waiting list	1.46 (0.96–2.22)	0.074		
Laboratory				
AST/ALT ratio	1.13 (1.03–1.24)	0.007		
Total bilirubin, mg/dL	1.03 (0.99–1.06)	0.095		
Albumin, g/dL	0.75 (0.53–1.05)	0.094		
Globulin, g/dL	0.92 (0.74–1.16)	0.491		
INR	3.73 (2.12–6.58)	< 0.001		
Creatinine, mg/dL	1.12 (1.03–1.22)	0.011		
Sodium, mmol/L	0.95 (0.90–0.99)	0.050		
Child–Turcotte–Pugh score	1.13 (1.02–1.24)	0.017		
MELD score	1.05 (1.01–1.09)	0.025		
MELD‐Na score	1.04 (1.01–1.07)	0.016	1.03 (1.00–1.07)	0.050
Sarcopenia	1.72 (1.12–2.66)	0.014	1.69 (1.04–2.76)	0.033

Abbreviations: ALT, alanine aminotransferase; AST, aspartate aminotransferase; BMI, body mass index; CI, confidence interval; INR, international normalized ratio; MELD, Model for End‐Stage Liver Disease; MELD‐Na, Model for End‐stage Liver Disease–sodium; sHR, subdistribution hazard ratio. Estimates were derived using the Fine–Gray competing risk model, treating liver transplantation as a competing event.

### 3.5. Clinical Outcomes Following Liver Transplantation

Among the 45 transplanted patients, the incidence of bacterial infection within the first 6 months posttransplant did not differ between patients with and without sarcopenia (11.1% vs. 11.1%, *p* = 1.000). Similarly, one‐year posttransplant mortality was comparable between sarcopenic and non–sarcopenic patients (11.1% vs. 16.7%, *p* = 0.684). The median intensive care unit (ICU) stay was 1 day (range, 1–8) versus 1 day [1–39] (*p* = 0.609). However, sarcopenic patients had a significantly longer hospital stay than non–sarcopenic patients (median 17 days [13–167] vs. 13.5 days [1–147], *p* = 0.034). Posttransplant outcomes were also similar between patients with HBV/HCV‐infected and nonviral‐related cirrhosis, including the incidence of bacterial infection within 6 months (13.8% vs. 16.2%, *p* = 0.446), 1‐year mortality (17.2% vs. 12.5%, *p* = 0.678), ICU stay (median 1 day [1–39] vs. 1 day [1–12], *p* = 0.165), and hospital length of stay (median 14 days [1–147] vs. 14 days [7–167], *p* = 0.943).

## 4. Discussion

Our study investigated the prognostic impact of sarcopenia in Thai cirrhotic patients awaiting liver transplantation. We found that 27.8% of patients had sarcopenia, as measured by L3‐SMI. These patients typically had lower BMI, serum albumin, and creatinine levels, along with more severe liver disease, as indicated by higher CTP scores. Sarcopenia was associated with shorter median transplant‐free survival, greater susceptibility to bacterial infections, and a higher risk of sepsis‐related death. Importantly, sarcopenia significantly increased the risk of death on the transplant waiting list, especially in patients with CTP class A/B cirrhosis. After adjusting for sex and MELD‐Na score, sarcopenia remained an independent predictor of waitlist mortality. These results highlight the urgent need for early identification and management of sarcopenia in cirrhotic patients to improve their outcomes while awaiting liver transplantation.

An ongoing issue regarding the prevalence of sarcopenia is the validity of the cutoff values for SMI across different regions and ethnicities. A recent meta‐analysis of 21 studies involving 6965 patients with cirrhosis reported a pooled prevalence of sarcopenia was 37.5% (95% CI 32.4%–42.8%) [8]. This analysis also included five Asian studies using L3‐SMI cutoffs to define sarcopenia: two from Korea, two from Japan, and one from China. In Korean studies, sarcopenia was defined as L3‐SMI ≤ 52.4 cm^2^/m^2^ for men and ≤ 38.5 cm^2^/m^2^ for women [32, 33], Japanese studies used cutoffs of ≤ 40.31 and ≤ 52.4 cm^2^/m^2^ for men and ≤ 30.88 and ≤ 38.5 cm^2^/m^2^ for women [34, 35], and a study from China defined sarcopenia as L3‐SMI ≤ 46.96 cm^2^/m^2^ for men and ≤ 32.46 cm^2^/m^2^ for women [36]. Notably, a single transplant center in the United States found significant SMI variations among cirrhotic patients awaiting liver transplantation, with median SMI highest in Black men (56 cm^2^/m^2^) and women (45 cm^2^/m^2^) and lowest in Asian men (46 cm^2^/m^2^) and women (36 cm^2^/m^2^) [37]. Studies show that Asians generally have lower skeletal muscle mass compared with Caucasians, resulting in varying prevalence rates of sarcopenia [9, 26, 27]. Based on Asian L3‐SMI values, our observation of a low sarcopenia prevalence aligned with previous reports ranging from 23.1% to 68.5% [32–36]. These findings highlight the broad range of proposed L3‐SMI cutoffs for sarcopenia across different racial or ethnic groups. Variations in SMI likely result from differences in muscle quality, genetic factors, hormone–environment interactions, socioeconomic factors like healthcare access, and physical activity rates. Therefore, there is a need for rigorously conducted prospective cohort studies to establish precise cutoffs for sarcopenia among diverse populations and to explore its correlation with hepatic decompensation and mortality in cirrhosis patients.

Our study found that sarcopenia was detected at similar rates among both male and female patients, which contrasts with existing literature that reports a higher prevalence of sarcopenia in males [38]. This discrepancy may be attributed to the relatively small proportion of female patients in our study population. Also, our findings showed no significant differences in sarcopenia prevalence among patients with cirrhosis of different etiologies. This is contrary to previous research indicating a higher association of sarcopenia with alcoholic cirrhosis compared with other causes [15, 39]. One possible explanation for our results is the predominance of chronic viral hepatitis as the leading cause of cirrhosis in our cohort, accounting for about 64% of the cases. In contrast, alcohol‐associated liver disease was less prevalent in our study, whereas it constitutes almost one‐third of the cases in the broader literature [8]. This difference in etiological distribution may have influenced the observed patterns of sarcopenia in our study.

Patients with sarcopenia had higher CTP scores but similar MELD and MELD‐Na scores compared with those without sarcopenia. This likely reflects the greater frequency of ascites in sarcopenic patients, which contributes to CTP but not MELD scoring. Notably, although the overall cohort had a near‐normal mean BMI, patients without sarcopenia fell within the overweight range based on Asian‐specific criteria (BMI ≥23 kg/m^2^), whereas those with sarcopenia had significantly lower BMI. Importantly, the higher prevalence of ascites in the sarcopenic group may have masked muscle loss by artificially maintaining body weight. These findings highlight that sarcopenia can occur independently of BMI in cirrhosis and underscore the limitations of BMI as a surrogate for nutritional status. In contrast, CT‐derived SMI provides a more reliable assessment of muscle mass and prognostic risk in this population.

Sarcopenia serves as an important adjunctive prognostic indicator in cirrhotic patients, adversely affecting multiple clinical outcomes. It has been consistently associated with an increased risk of infectious complications in cirrhosis [40]. Proposed mechanisms include impaired immune competence due to reduced skeletal muscle mass, altered amino acid availability, and gut dysbiosis with increased bacterial translocation, all of which contribute to heightened susceptibility to infection [41–45]. Consistent with these observations, our study showed that patients with sarcopenia had a higher incidence of bacterial infections and were more likely to die from sepsis than those without sarcopenia, underscoring the importance of addressing sarcopenia to improve outcomes. Early detection of sarcopenia is therefore critical, as targeted prehabilitation may help preserve functional reserve in patients awaiting liver transplantation [46, 47]. Recommended strategies include individualized nutritional optimization, particularly protein intake > 1.5 g/kg/day with frequent meals and a late‐evening snack, and structured exercise programs tailored to clinical status [47, 48]. These interventions are generally safe and feasible, with evidence supporting improvements in muscle mass, strength, and functional capacity, which may enhance readiness for transplantation [46].

Sarcopenia holds significant prognostic value among cirrhotic patients awaiting liver transplantation, affecting both survival and posttransplant outcomes [18, 21, 47, 49–51]. In the present study, sarcopenia was independently associated with increased waitlist mortality. Exploratory subgroup analyses suggested that this association was more pronounced in patients with HBV/HCV‐related cirrhosis than in those with nonviral‐related cirrhosis. Additional exploratory analyses within the HBV/HCV subgroup demonstrated similar trends according to the presence of coexisting alcohol‐related or metabolic features. However, these findings should be interpreted cautiously, as formal interaction analyses were not statistically significant. The disproportionate impact of sarcopenia observed in patients with viral hepatitis–related cirrhosis may suggest that muscle depletion reflects more advanced systemic injury in this subgroup [52]. One possible explanation is that, despite virologic suppression, persistent immune dysregulation and low‐grade inflammation may continue to contribute to muscle proteolysis and impaired muscle synthesis. Existing literature suggests that direct‐acting antiviral‐induced HCV clearance may not completely normalize inflammatory cytokine and chemokine profiles [53]. However, inflammatory markers and longitudinal viral suppression status were not directly assessed in the present study. Therefore, these observations remain exploratory and hypothesis‐generating, and further prospective studies incorporating longitudinal viral load data and inflammatory biomarker assessments are needed to clarify the underlying mechanisms.

In our transplanted cohort, sarcopenia did not influence early posttransplant infections or short‐term mortality but was associated with prolonged hospitalization, indicating increased postoperative resource utilization. Posttransplant outcomes were otherwise comparable across etiologies, supporting the dominant role of perioperative factors over underlying disease in early recovery. These findings are consistent with evidence that CT‐derived sarcopenia is a robust predictor across the transplant continuum, particularly for healthcare utilization [54]. Given our sample size, larger studies are warranted to further investigate how the achievement of viral control interacts with preexisting sarcopenia to determine long‐term survival.

The clinical implications of our findings are notable. First, our findings highlight the importance of early identification and management of sarcopenia prior to transplantation to improve outcomes, particularly in Asian populations where its impact on mortality may be more pronounced [23]. Consistent with prior studies, sarcopenia appears to exert a greater prognostic impact in compensated and early decompensated cirrhosis, with diminishing influence in advanced disease [8, 16, 32, 47, 55]. In our cohort, sarcopenia significantly increased waitlist mortality in patients with CTP class A/B cirrhosis but not in those with CTP class C, likely reflecting the dominant effect of severe hepatic dysfunction in advanced stages. These findings highlight the importance of early detection and proactive management of sarcopenia before progression to advanced decompensation. Second, our results challenge the “sickest first” approach in organ allocation. Incorporating sarcopenia into allocation models, such as a MELD‐sarcopenia score, may enhance risk stratification by identifying high‐risk patients not fully captured by MELD‐Na. However, its implementation as an exceptional listing criterion requires standardized assessment, population‐specific cutoffs, and prospective multicenter validation. Further studies in larger cohorts, particularly in advanced cirrhosis, are needed to better define its prognostic role.

Our study had several notable strengths. We utilized CT‐based SMI assessment at the L3 level, which has been widely adopted as a practical and reliable method for evaluating muscle mass in cirrhotic patients awaiting liver transplantation [7, 9, 21]. Although dual‐energy X‐ray absorptiometry is commonly used to assess muscle mass in the general population, its accuracy may be limited in cirrhosis due to the presence of ascites and fluid retention. In contrast, CT‐derived SMI minimizes these confounding factors and is routinely available as part of transplant evaluation protocols, supporting its feasibility and clinical relevance in this specific population [9]. Notably, all muscle measurements were conducted by an experienced radiologist blinded to clinical and laboratory data, ensuring objectivity and consistency. In addition, the use of competing risk analysis allowed for appropriate adjustment for liver transplantation as a competing event, improving the precision of mortality risk estimation. Finally, our study provides valuable data from an Asian cohort and offers region‐specific insights into the prognostic role of sarcopenia in cirrhosis.

This study has several limitations. First, the retrospective design restricted the assessment of sarcopenia to CT‐derived L3‐SMI alone, and functional parameters such as handgrip strength or gait speed were unavailable. This may have resulted in some degree of misclassification and potentially underestimated the true prevalence of sarcopenia. However, L3‐SMI remains the most commonly applied modality for skeletal muscle mass assessment in transplant candidates, particularly in retrospective studies [8, 21]. Second, patient management was not protocolized, which may have introduced variability in clinical decision‐making, including the initiation of antibiotic therapy or nutritional interventions, potentially influencing patient outcomes. In addition, sarcopenia‐directed interventions were not standardized and were inconsistently documented in this retrospective study, precluding evaluation of their effects on clinical outcomes. Lastly, the study was conducted at a single transplant center in Thailand, which may limit generalizability to other healthcare settings. However, the use of standardized transplant evaluation protocols and uniform imaging techniques enhances the internal validity of our findings and offers meaningful insight into sarcopenia within an Asian population, where evidence is relatively scarce.

## 5. Conclusion

This study demonstrates that sarcopenia plays a significant prognostic role in cirrhotic patients awaiting liver transplantation. Using CT‐based assessment of skeletal muscle mass, we quantified the burden of sarcopenia and identified its association with higher waitlist mortality and increased risk of infectious complications. These results emphasize the need for routine evaluation of muscle mass and nutritional status in transplant candidates. Early identification of sarcopenia may allow timely implementation of supportive measures, including nutritional optimization, physical rehabilitation, ammonia‐reducing strategies, and microbiota‐focused therapies, which could potentially improve clinical outcomes. Further prospective studies are warranted to confirm these findings and to assess whether targeted interventions can enhance survival before and after transplantation.

## Author Contributions

All authors (Sorachat Niltwat, Pornpim Korpraphong, Khemajira Karaketklang, and Phunchai Charatcharoenwitthaya) significantly contributed to the presented work, encompassing conceptualization, study design, implementation, data collection, analysis, and interpretation. In addition, this article was drafted, revised, and critically evaluated by Phunchai Charatcharoenwitthaya. All authors approved the final version of this paper for publication and selected the journal for submission. Furthermore, they had full responsibility for all parts of the work and agreed to be accountable for the article′s content and conclusions.

## Funding

No funding was received for this manuscript.

## Ethics Statement

This study was approved by the Institutional Review Board of the Faculty of Medicine Siriraj Hospital (COA no. Si547/2013) and was performed according to the Declaration of Helsinki. Informed consent was waived by the Ethics Committee due to the retrospective design of the study.

## Consent

The authors have nothing to report.

## Conflicts of Interest

The authors declare no conflicts of interest.

## Data Availability

The data that support the findings of this study are available on request from the corresponding author. The data are not publicly available due to privacy or ethical restrictions.
